# Revealing the influence of steric bulk on the triplet–triplet annihilation upconversion performance of conjugated polymers

**DOI:** 10.1038/s41598-021-99179-y

**Published:** 2021-10-01

**Authors:** Riley O’shea, William J. Kendrick, Can Gao, Tze Cin Owyong, Jonathan M. White, Kenneth P. Ghiggino, Wallace W. H. Wong

**Affiliations:** 1 School of Chemistry, ARC Centre of Excellence in Exciton Science, University of Melbourne, Parkville, VIC 3010 Australia; 2 School of Chemistry, Bio21 Institute, University of Melbourne, Parkville, VIC 3010 Australia; 3 Beijing National Laboratory for Molecular Sciences, Key Laboratory of Organic Solids, Institute of Chemistry, Chinese Academy of Sciences, Beijing, China

**Keywords:** Optical materials, Conjugated polymers, Light harvesting, Energy transfer

## Abstract

A series of poly(phenylene-vinylene)-based copolymers are synthesized using the Gilch method incorporating monomers with sterically bulky sidechains. The photochemical upconversion performance of these polymers as emitters are investigated using a palladium tetraphenyltetrabenzoporphyrin triplet sensitizer and MEH-PPV as reference. Increased incorporation of sterically bulky monomers leads to a reduction in the upconversion efficiency despite improved photoluminescence quantum yield. A phosphorescence quenching study indicates issues with the energy transfer process between the triplet sensitizer and the copolymers. The best performance with 0.18% upconversion quantum yield is obtained for the copolymer containing 10% monomer with bulky sidechains.

## Introduction

**Figure 1:**
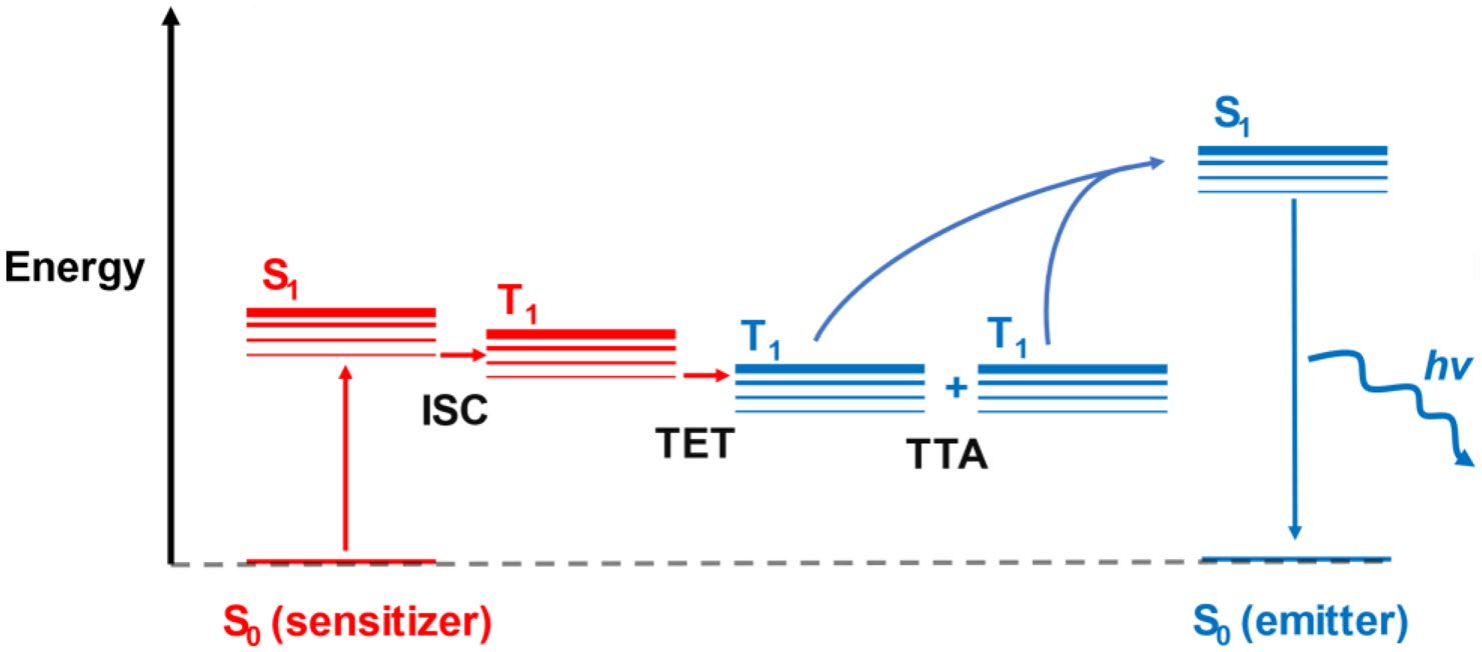
Jablonski diagram depicting the processes involved in TTA-UC with a sensitizer-emitter pair.

Triplet–triplet annihilation upconversion (TTA-UC), also known as triplet fusion, is a photochemical process by which two lower energy photons can be used to produce one photon of higher energy^1^. It sees use in raising the efficiency of solar cells above their thermodynamic limit (the Shockley-Queisser limit)^2,3^. To achieve efficient TTA-UC, several photochemical processes must effectively take place, which are often performed by a two-component system, with a “triplet sensitizer” and “emitter” (Fig. 1). The role of the triplet sensitizer is to absorb incoming light and efficiently generate the triplet excited state via intersystem crossing (ISC). The triplet excitons generated may then be transferred to an emitter molecule to generate the triplet excited state of the emitter through triplet energy transfer (TET). Two emitters in their triplet excited states may then annihilate, allowing one emitter to return to the ground state and the other emitter to preferably form the singlet excited state (TTA). The emitter molecule can then produce a photon with higher energy than the original incident photon used to excite the sensitizer.

The upconversion quantum yield (*Φ*_UC_) is a product of all the quantum yields for photochemical processes involved, given by the equation:

1 $${\Phi }_{\text{UC}}={\Phi }_{\text{ISC}}{\Phi }_{\text{TET}}{\Phi }_{\text{TTA}}{\Phi }_{\text{PL}},$$

where *Φ*_ISC_ is the ISC quantum yield, *Φ*_TET_ is the TET quantum yield, *Φ*_TTA_ is the TTA quantum yield, and *Φ*_PL_ is the photoluminescence quantum yield^4^. *Φ*_TTA_ can be further separated into the contact triplet pair formation efficiency and the factor *f*^5^, which is a measure of the probability of generating the singlet excited state after TTA, since singlet, triplet and quintet states are all possible outcomes. This factor may differ significantly between different emitters^6–9^.

Typically, in TTA upconversion systems, only small molecule emitters are used in conjunction with transition metal porphyrin-based triplet sensitizers. Many of the most efficient upconversion systems use platinum(II) octaethylporphyrin (PtOEP) as the triplet sensitizer and 9,10-diphenylanthracene (DPA) as the emitter or derivatives thereof^10–12^. Very few examples exist of polymer-based emitters. They typically consist of molecular emitter chromophores pendant on polymeric backbones^11,13–16^. In these studies, the aim was to create condensed or solid-state systems where the TTA upconversion processes were not dependent on molecular diffusion. Unfortunately, the covalently linkage of the chromophores negatively impacted the upconversion performance. The reasons for this include lower *Φ*_*PL*_ of the emitter, lower TET rate and/or efficiency, shorter triplet lifetimes and lower TTA efficiency. In some cases, the covalently linked chromophores did not even perform as well as systems where chromophores are simply dispersed in polymer matrices^17–19^.

**Figure 2:**
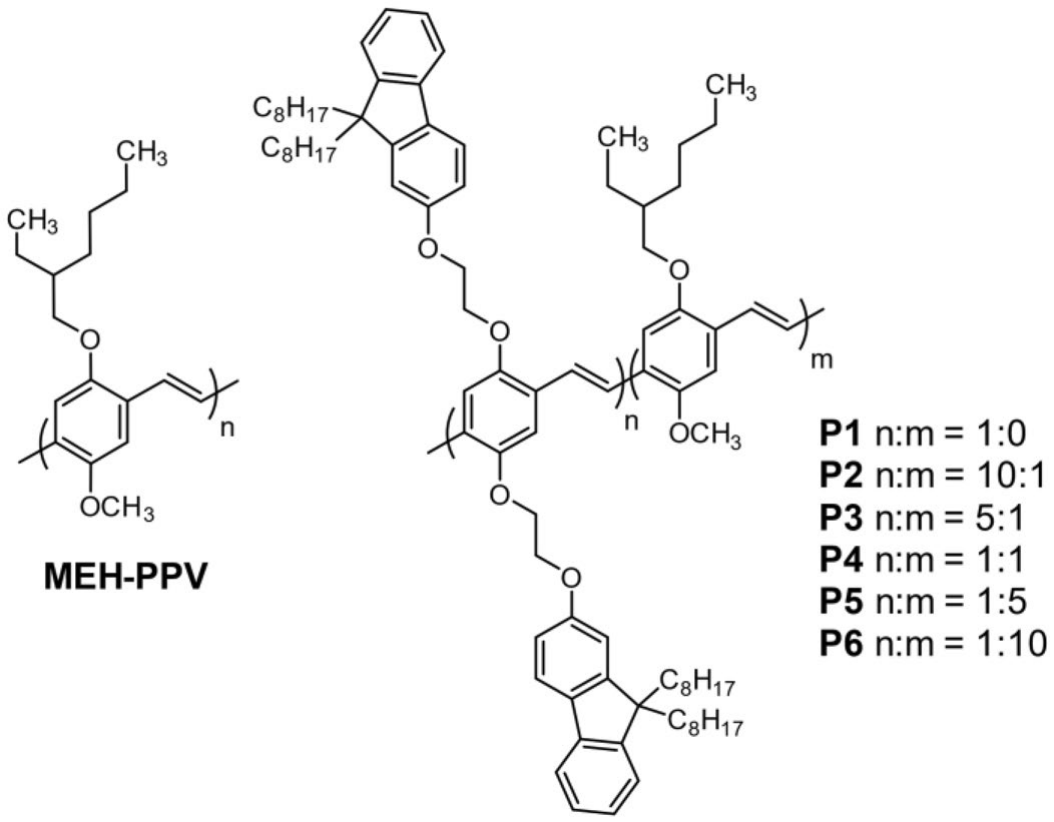
Structures of MEH-PPV and the proposed PPV copolymers **P1–P6** as emitters.

Even fewer studies used conjugated polymers as emitters^20–24^. Polyfluorenes, with either simple alkyl side-chains^20^, a spiro-bisfluorene motif^21^, or aryl side-chains on a fused polyfluorene^22,23^, have been reported. Another example of a conjugated polymer emitter shown by Monkman and co-workers used a commercially available poly(phenylene-vinylene) (PPV) copolymer with the trade name “Super yellow PPV” with palladium(II) tetraphenyltetrabenzoporphyrin (PdTPTBP) as the triplet sensitizer^24^. The use of PPV emitters over other conjugated polymers may be particularly advantageous owing to their high singlet–triplet excited state energy gap^25^. Additionally, Lyskov et al. have shown that the triplet exciton transport may proceed via a fast intramolecular diffusion along the PPV backbone^26^, not possible for small molecule emitters. This improvement of triplet exciton transport may not necessarily be limited to PPVs but is expected to be a general property of conjugated polymers with planar geometry. In this study, a series of PPV-based copolymers were synthesized, characterized and their upconversion performance as emitters measured. The primary objective was to investigate the effect of increasing composition of monomers with bulky sidechains starting with the well-known poly[2-methoxy-5-(2-ethylhexyloxy)-1,4-phenylenevinylene] (MEH-PPV). The proposed PPV copolymers **P1–P6** have fluorenyloxy side chains with an ethylene glycol spacer to decouple the electronic interactions between the fluorenyl unit and the polymer backbone (Fig. 2). The dioctylfluorene units are introduced to insulate the polymer backbone, such that high *Φ*_*PL*_ can be achieved by reducing aggregation-induced quenching.

## Methods

### Materials

Commercial reagents were purchased from Univar, Sigma-Aldrich, AK Scientific, Matrix Scientific, Ajax Finechem, and Labchem, and were used as received. The reference MEH-PPV polymer was purchased commercially from Lumtec (https://lumtec.com.tw/). Anhydrous toluene, diethyl ether, dichloromethane, and tetrahydrofuran were obtained from alumina drying columns. For reactions carried out under inert conditions, standard Schlenk techniques were used. Solvents were sparged with nitrogen gas for several hours prior to use, and the reaction vessels were sealed with a rubber septum under a nitrogen atmosphere. Thin layer chromatography (TLC) was done using Merck-Millipore Silica gel glass plates (60G F254), with a 254 nm and 365 nm light mercury lamp used for identifying spots. ^1^H NMR (400 MHz) and ^13^C NMR (100 MHz) spectra were obtained in CDCl_3_, on a 400 MHz Varian spectrometer. NMR peaks were referenced to the CHCl_3_ solvent peak. UV–Vis spectroscopy was performed on an Agilent Technologies Cary 50 UV–Vis, using an L = 0.01 cm cell to account for the high concentration of the samples. Photoluminescence spectroscopy was performed on a Varian Cary Eclipse fluorimeter using a triangular cell in a front-facing fluorescence experiment to minimize sample reabsorption. Absolute photoluminescence quantum yield of the samples was determined via an integrating sphere method using an integrating sphere accessory (F3018, Horiba Jobin Yvon) on a Fluorolog-3 fluorimeter (see Supplementary Information for Experimental Details).

### Synthesis procedures

#### Compound 2

9,9-Dioctyl-9H-fluoren-2-ol **1** (3.8 g, 9.34 mmol), ethylene carbonate (1.9 g, 21.6 mmol), potassium carbonate (4 g, 28.9 mmol), and tetrabutylammonium bromide (0.4 g, 1.2 mmol) were dissolved in 80 ml of DMF and heated to 110 °C overnight under nitrogen atmosphere. The mixture was then cooled and poured into water. The product was then extracted with ethyl acetate and washed with 5% w/v aqueous lithium chloride. The organic layer was then collected and diluted with hexanes and dried over magnesium sulfate. The mixture was then filtered through a thin layer of silica, then the solvent was evaporated. Mass = 4.2 g (quant.).

^1^H NMR (400 MHz, CHCl_3_, δ): 7.61 (m, 2H), 7.28 (m, 2H), 7.23 (m, 1H), 6.89 (m, 2H), 4.15 (t, *J* = 4.5Hz, 2H), 4.01 (t, *J* = 4.2Hz, 2H), 2.06 (s, 1H), 1.91 (m, 4H), 1.11 (m, 21H), 0.82 (t, *J* = 7.1Hz, 6H), 0.62 (m, 4H); ^13^C NMR (100 MHz, CDCl_3_, δ):158.50, 152.75, 150.20, 140.91, 134.64, 126.67, 125.98, 122.68, 120.34, 118.86, 112.70, 109.56, 69.40, 61.61, 55.04, 40.53, 31.77, 30.03, 29.22, 23.69, 22.58, 14.05; HRMS (ESI) *m*/*z*: [M]^+^ calcd for C_31_H_46_O_2_, 450.3498; found, 450.3498.

#### Compound 3

Triphenylphosphine (2.44 g, 9.3 mmol) was dissolved in 15 ml of DCM and cooled to 0 °C. A solution of *N*-bromosuccinimide (1.67 g, 9.3 mmol) in 15 ml of DCM was added to the mixture dropwise. Then (2-((9,9-dioctyl-9H-fluoren-2-yl)oxy)ethan-1-ol **2** (4.2 g, 9.3 mmol) in 15 ml of DCM was added to the mixture dropwise. The mixture was then poured into hexanes and filtered through a thin layer of silica. The solvent was then evaporated, and the product was loaded onto celite. The product was then purified by DCVC eluting with 0, 0.5, 1, 2, 3% ethyl acetate in hexanes. Mass = 3.5 g (73%)

^1^H NMR (400 MHz, CHCl_3_, δ): 7.61 (m, 2H), 7.29 (m, 2H), 7.24 (m, 1H), 6.88 (m, 2H), 4.36 (t, *J* = 6.3Hz, 2H), 3.68 (t, *J* = 6.3Hz, 2H), 1.91 (m, 4H), 1.11 (m, 21H), 0.82 (t, *J* = 7.1Hz, 6H), 0.61 (m, 4H); ^13^C NMR (100 MHz, CDCl_3_, δ):157.96, 152.80, 150.23, 140.82, 134.94, 126.68, 126.06, 122.70, 120.37, 118.91, 112.96, 109.85, 68.20, 55.07, 40.47, 31.76, 30.00, 29.22, 29.21, 29.20, 23.67, 22.58, 14.05; HRMS (ESI) *m*/*z*: [M]^+^ calcd for C_31_H_45_OBr, 512.2654; found, 512.2655.

#### Compound 5

2-(2-Bromoethoxy)-9,9-dioctyl-9H-fluorene **3** (3.5 g, 6.8 mmol), dimethyl 2,5-dihydroxyterephthalate **4** (0.62 g, 2.74 mmol), potassium carbonate (1.9 g, 13.7 mmol), and tetrabutylammonium bromide (0.56 g, 1.68 mmol) were dissolved in 50 mL of DMF and heated to 110 °C overnight under nitrogen atmosphere. The mixture was then cooled and poured into water. The product was then extracted with chloroform and washed with 5% w/v aqueous lithium chloride. The organic layer was then collected and dried over magnesium sulfate. The mixture was then filtered through a thin layer of silica, then the solvent was evaporated, and the product was loaded onto celite. The product was then purified by DCVC eluting with 0, 1, 2, 5, 10, 20% ethyl acetate in hexanes. Mass = 2.01 g (67%)

^1^H NMR (400 MHz, CHCl_3_, δ): 7.62 (m, 6H), 7.31 (m, 4H), 7.24 (m, 2H), 6.95 (m, 4H), 4.45 (m, 8H), 3.90 (s, 6H), 1.95 (m, 8H), 1.12 (m, 45H), 0.84 (t, *J* = 7.1Hz, 12H), 0.65 (m, 8H); ^13^C NMR (100 MHz, CDCl_3_, δ): 165.73, 158.56, 152.74, 152.36, 150.22, 140.96, 134.65, 126.70, 125.99, 125.36, 122.69, 120.34, 118.89, 118.45, 112.84, 109.75, 69.44, 66.90, 55.07, 52.36, 40.57, 31.79, 30.08, 29.26, 23.75, 22.60, 14.08; HRMS (ESI) *m*/*z*: [M]^+^ calcd for C_72_H_98_O_8_, 1090.7262; found, 1090.7262.

#### Compound 6

Lithium aluminium hydride (0.724 g, 19 mmol) was dissolved in 40 mL of diethyl ether and cooled to 0 °C. A solution of dimethyl 2,5-bis(2-((9,9-dioctyl-9H-fluoren-2-yl)oxy)ethoxy)terephthalate **5** (2.01 g, 1.84 mmol) in 20 ml of diethyl ether was added dropwise to the mixture. The mixture was then allowed to slowly warm to room temperature overnight. The mixture was then cooled to 0 °C, and 0.7 ml of water was added dropwise to the mixture, followed by 0.7 ml of 1 M aqueous sodium hydroxide, and finally another 2.1 ml of water. The mixture was then stirred at room temperature for 15 min. Then 4 g of magnesium sulfate was added, and the mixture was stirred for another 15 mins. The mixture was then filtered through a thin layer of silica. The solvent was evaporated, and the product was loaded onto celite. The product was then purified by DCVC eluting with 0, 5, 10, 20% ethyl acetate in hexanes. Mass = 1.91 g (quant).

^1^H NMR (400 MHz, CHCl_3_, δ): 7.60 (m, 4H), 7.28 (m, 4H), 7.24 (m, 2H), 6.97 (s, 2H), 6.61 (m, 4H), 4.71 (s, 4H), 4.40 (m, 8H), 1.92 (m, 8H), 1.12 (m, 45H), 0.82 (t, *J* = 7.1Hz, 12H), 0.62 (m, 8H); ^13^C NMR (100 MHz, CDCl_3_, δ): 158.30, 152.82, 151.10, 150.24, 140.87, 134.80, 130.42, 126.67, 126.00, 122.71, 120.42, 118.91, 113.89, 112.99, 109.39, 68.15, 66.78, 62.03, 55.09, 40.46, 31.77, 30.03, 29.23, 29.21, 23.71, 22.58, 14.06; HRMS (ESI) *m*/*z*: [M]^+^ calcd for C_70_H_98_O_6_, 1034.7363; found, 1034.7362.

#### Compound 7

(2,5-Bis(2-((9,9-dioctyl-9H-fluoren-2-yl)oxy)ethoxy)-1,4-phenylene)dimethanol **5** (1.91 g, 1.84mmol) and 0.34 ml of pyridine were dissolved in 20 ml of chloroform and cooled to 0 °C. A solution of thionyl chloride (0.43 ml) in 9 ml of chloroform was added dropwise to the mixture. The mixture was then allowed to slowly warm to room temperature and stirred for 2 h. The mixture was then poured into saturated aqueous sodium bicarbonate. The product was then extracted with chloroform and then the organic layer was collected and dried over magnesium sulfate. The mixture was then filtered through a thin layer of silica and the solvent was evaporated. The mixture was stirred in hexanes and filtered. The product was then washed with cold hexanes. Mass = 1.54 g (78%)

^1^H NMR (400 MHz, CHCl_3_, δ): 7.61 (d, *J* = 8.5Hz, 4H), 7.30 (m, 4H), 7.23 (m, 2H), 7.08 (s, 2H), 6.93 (m, 4H), 4.69 (s, 4H), 4.41 (s, 8H), 1.93 (m, 8H), 1.11 (m, 44H), 0.82 (t, *J* = 7.1Hz, 12H), 0.63 (m, 8H); ^13^C NMR (100 MHz, CDCl_3_, δ): 158.53, 152.79, 150.83, 150.25, 140.92, 134.67, 127.94, 126.67, 125.97, 122.70, 120.34, 118.88, 115.40, 112.82, 109.78, 68.52, 66.89, 55.06, 41.08, 40.50, 31.77, 30.04, 29.23, 29.22, 23.72, 22.58, 14.06; HRMS (ESI) *m*/*z*: [M]^+^ calcd for C_70_H_96_O_2_Cl_2_, 1070.6686; found, 1070.6686.

### General polymerization method

Potassium tert-butoxide (20 eq.) was dissolved in dry degassed THF (made to 0.4 M) and stirred at room temperature under nitrogen atmosphere. Then the monomer(s) (1 eq.) were added dropwise as a solution of dry degassed THF (made to 0.035 M) to the mixture. The mixture was then allowed to stir overnight at room temperature. The mixture was then diluted with THF and poured into 10 times the reaction volume of methanol with vigorous stirring. The suspension was then centrifuged to collect the polymer, which was then washed 3 times with methanol and then dried in a vacuum oven overnight. Example procedure for **P4** provided below. See Table S2 in the Supporting Information file for synthesis details for **P1** to **P6**. GPC traces are shown in Fig. S17.

#### Example procedure: synthesis of P4

Potassium tert-butoxide (0.317 g) was dissolved in 7 ml of dry degassed THF and stirred at room temperature under nitrogen atmosphere. Then 2,2ʹ-((((2,5-bis(chloromethyl)-1,4-phenylene)bis(oxy))bis(ethane-2,1-diyl))bis(oxy))bis(9,9-dioctyl-9H-fluorene) **7** (0.114 g, 0.106 mmol) and 1,4-bis(bromomethyl)-2-((2-ethylhexyl)oxy)-5-methoxybenzene **8** (0.045 g, 0.106 mmol) were added dropwise as a solution in 4 ml of dry degassed THF to the mixture. The mixture was then allowed to stir overnight at room temperature. The mixture was then diluted with THF and poured into 110 ml of methanol with vigorous stirring. The suspension was then centrifuged to collect the polymer, which was then washed 3 times with methanol and then dried in a vacuum oven overnight. Mass = 0.084 g (63%)

### Upconversion experiments

Upconversion samples were degassed three times via freeze-pump-thaw cycles prior to analysis. Upconversion spectra were taken using an Ocean Optics USB spectrometer with 300 μm fiber optic cable.

The upconversion quantum yields were measured using the following equation:

2 $${\Phi }_{UC}={\Phi }_{Ref}\left(\frac{{A}_{Ref}}{{A}_{Unk}}\right)\left(\frac{{I}_{Unk}}{{I}_{Ref}}\right){\left(\frac{{\eta }_{Unk}}{{\eta }_{Ref}}\right)}^{2},$$

using a degassed solution of MEH-PPV (0.5 mg/mL) with PdTPTBP (7.5 μM) in chloroform as the reference material. The ${\Phi }_{UC}$ of the MEH-PPV standard material was determined to be 0.039% with 632 nm excitation (band pass filtered HeNe laser) with an intensity of 985 mW/cm^2^ via an integrating sphere method with a LABSPHERE (model number: 4P-GPS-053-SL) and detected with a liquid nitrogen cooled CCD camera from Princeton Instruments (series number: SP2500). Band pass filters for both short and long pass filters were obtained from Thorlabs (Edgepass filter range). This was used as the reference (*Φ*_*Ref*_) for all other values given.

The values for the triplet energy transfer quantum yields were determined by comparing the integrated emission from the sensitizer with/without the polymer present, using the following equation:

3 $${\Phi }_{\text{TET}}=1-\frac{\int {I}_{polymer}\left(\lambda \right)d\lambda }{\int {I}_{blank}\left(\lambda \right)d\lambda },$$

where I_polymer_ represents the spectra of the sample with the polymer present and I_blank_ is the sample without the polymer.

## Results

### Synthesis and characterization

**Figure 3:**
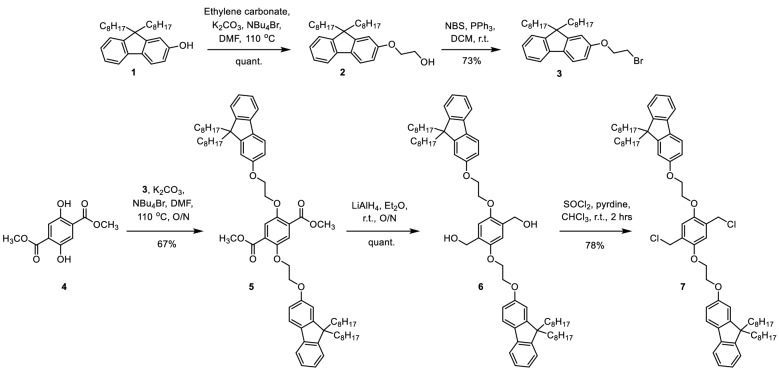
Synthesis of monomer **7** starting from dioctylfluorenol.

**Figure 4:**
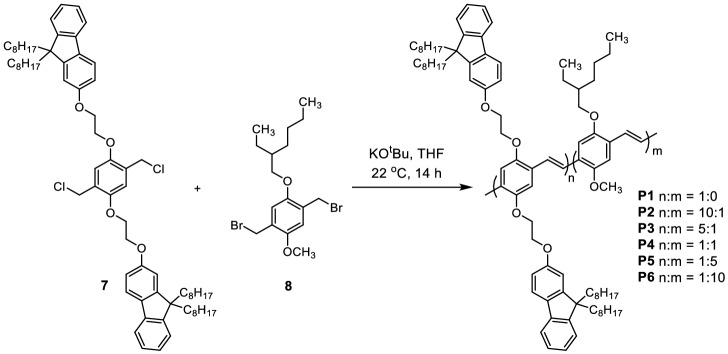
Gilch polymerization with monomer 7 and the MEH monomer 8 to produce the PPV copolymer **P1–P6**.

The synthesis of the new PPV copolymers first began by alkylation of dioctylfluorenol **1** with ethylene carbonate to install the glycol spacer **2** in a quantitative yield (Fig. 3). The alcohol **2** was then converted to the corresponding bromide **3** in a 73% yield. The bromide **3** was installed via an alkylation onto the methyl ester terephthalate core **4** in a 67% yield. The methyl ester **5** were reduced with lithium aluminium hydride to produce the corresponding alcohol **6** in a quantitative yield. Monomer **7** was produced in a 78% yield by treating the alcohol **6** with thionyl chloride and pyridine. Attempts made using thionyl chloride in the absence of pyridine failed to produce the desired product, due to the susceptibility of the glycol unit to acidic conditions, this property made purification via column chromatography difficult. The single crystal structure of monomer **7** was then obtained from slowly cooling a saturated solution in DMF. The structure showed an unusual kink in one of the octyl chains on each fluorene as it packed closely with neighbouring units (For more details see Table S1, Fig. S16). However, the fluorene units remained distant from the phenylene core ensuring electronic decoupling with the polymer backbone as desired. The PPV copolymers **P1–P6** were then prepared by Gilch polymerization^27^ in THF with various ratios of monomer **7** and the MEH monomer **8** (Fig. 4). Reaction yields ranged between 63 and 99% (Table S2). It is important to note here that the exact monomer ratio incorporated into the polymers could not be determined using standard methods such as NMR, UV–Vis absorption or photoluminescence spectroscopy. The NMR signals are broad as expected for polymers and there were no distinct chemical shifts for the monomers that allow integration of the signals. Estimates from either the UV–Vis absorbance intensities or fluorescence peak emission shift leads to unrealistic values. This may be due to the nature of the statistically random copolymerization which may form a range of different chromophores each with their own extinction coefficients with varying photoluminescent quantum yields. The monomer feed ratios (n:m) were not expected to exactly match the true copolymer ratios, since the Gilch polymerization produced a statistically random polymer. However, a sufficiently large enough range of comonomer ratios ensured that trends in properties were maintained.

Having successfully synthesized the PPV copolymers **P1–P6**, their general and photophysical properties were determined. Comparing the data for all the synthesized copolymers (Table 1), the number-average molecular weight (M_n_) spanned a relatively wide range, differing by a factor of six between the lowest and highest values. However, due to the stark difference in molecular weight of the repeat units derived from monomers **7** and **8** (1072.43 and 422.20 g/mol, respectively), it was more useful to calculate and compare the average chain lengths in number of phenylenevinylene repeat units instead. This provided the average number of chromophores in a chain, as shown below. The chromophore length was previously determined for PPVs at 11 repeat units by Kolshorn et al.^28^. It was clear that most polymer samples had similar number-average chain lengths, though the samples with n:m = 5:1 and 1:10 were noticeable outliers, differing by roughly a factor of two from the rest.

**Table 1:** Molecular weight data (weight-average molecular weight, *M*_*w*_ number-average molecular weight, *M*_*n*_ and dispersity (Ɖ) of PPV copolymers P1–P6 and MEH-PPV reference material and upconversion performance parameters of samples containing these polymer emitters.

Polymer	n:m	M_w_ (g/mol)	M_n_ (g/mol)	Ɖ	Degree of polymer-isation^a^	Average # of chromophores^b^	*Φ*_PL_ (%)^c^	*Φ*_UC_ (%)^d^	*Φ*_TET_ (%)^e^	*Φ*_TTA_ (%)^f^
**P1**	1:0	450,000	95,100	4.7	95	8	58 ± 3	0.0087	1.0	1.50
**P2**	10:1	483,000	70,800	3.6	76	7	50 ± 7	0.011	10	0.22
**P3**	5:1	452,000	193,000	2.5	220	20	51 ± 3	0.020	19	0.21
**P4**	1:1	144,000	39,000	3.7	62	6	43 ± 1	0.081	51	0.37
**P5**	1:5	142,000	31,600	4.5	82	7	36 ± 5	0.150	57	0.73
**P6**	1:10	163,000	59,100	2.8	181	16	37 ± 4	0.160	63	0.69
MEH-PPV	–	62,500	20,100	3.1	77	7	30 ± 3	0.052	87	0.20

^a^Degree of polymerisation determined from the ratio of M_n_ and the average monomer molecular weight based on the initial stoichiometry used in the polymerisation.

^b^Calculated based on the degree of polymerisation and assuming 11 repeat units per chromophore^28^.

^c^*Φ*_PL_ of **P1–P6** (0.25 mg/ml) and MEH-PPV (0.5 mg/ml) measured in chloroform with excitation at 440 nm (see Supplementary Information for experimental details), error given as 2σ (N = 3).

^d^*Φ*_UC_ of **P1–P6** (0.25 mg/ml) and MEH-PPV (0.5 mg/l with PdTPTBP (7.5 μM) as the sensitizer in chloroform solution using 632 nm wavelength and 10,468 mW/cm^2^ power excitation.

^e^*Φ*_TET_ was obtained using the integrated phosphorescence intensity of the copolymer samples compared with that of the sensitizer-only sample (see Supplementary Information for Experimental Details).

^f^*Φ*_TTA_ was calculated using Eq. (1).

**Figure 5:**
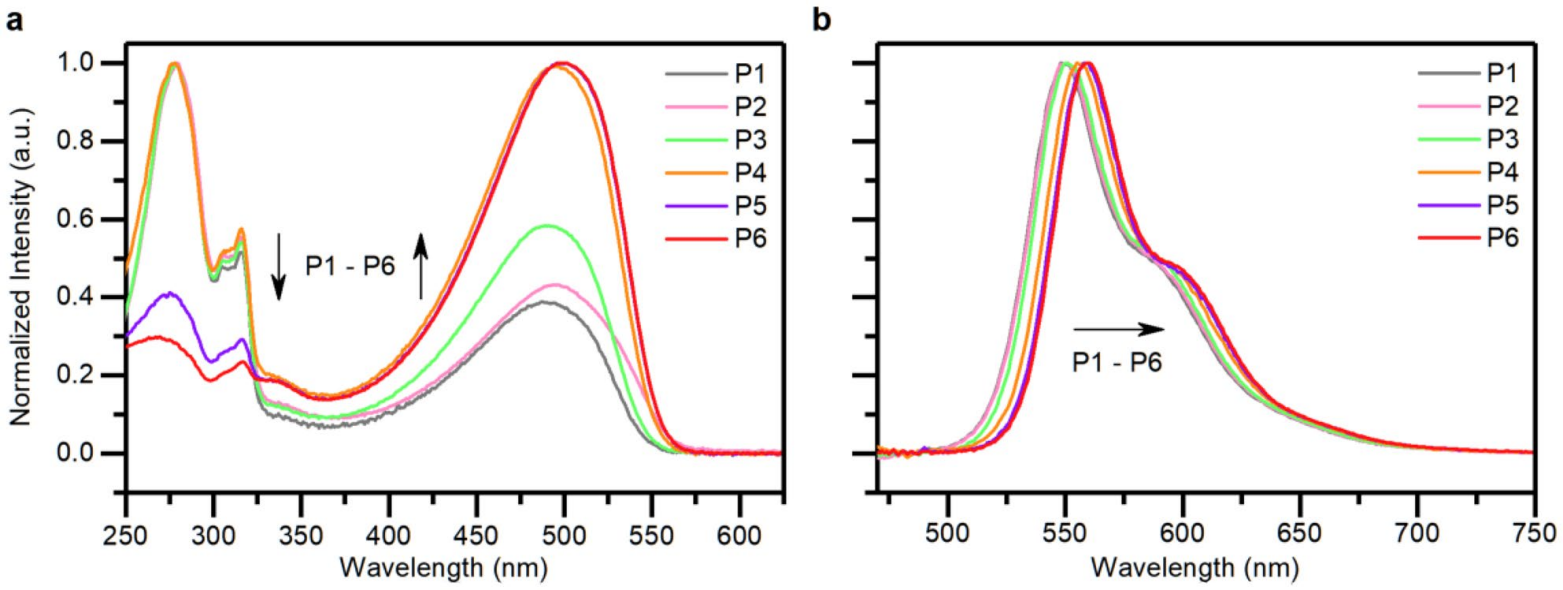
Normalized UV–Vis absorption spectrum (**a**) and photoluminescence spectrum with 450 nm excitation wavelength (**b**) of PPV copolymers **P1–P6** at 0.25 mg/ml in chloroform.

The UV–Vis absorption spectrum of the polymers showed peaks at 278, 316 and 497 nm with significant absorbance variation amongst the 6 samples (Fig. 5a). The absorbance variation was a result of the varying proportion of the fluorenyloxy comonomer unit. The peaks at 316 nm and 278 nm were attributed to the fluorene itself, and for all comonomer ratios except 1:5 and 1:10 (**P5** and **P6** respectively) the relative intensity of the fluorenyl band was greater than or equal to that of the PPV polymer backbone (λ = 497 nm). This was primarily due to a greater abundance of fluorenyl units compared to effective polymeric chromophores. There was only a small shift in the peak maxima of the photoluminescence spectrum for this series of PPV copolymers (Fig. 5b). The peak maxima varied from 548 nm for **P1** (n:m = 1:0) to 560 nm for **P6** (n:m = 1:10). This could be due to a slight twisting of the polymer backbone caused by steric repulsion of the fluorenyloxy side chains reducing effective conjugation, an effect which diminishes as the content of the MEH monomer increases. The *Φ*_PL_ of the polymers (Table 1) show a convincing correlation to the proportion of the bulky fluorenyloxy comonomer unit in the polymers (see Supplementary Information, Fig. S23) with a decrease in the amount of the bulky fluorenyloxy comonomer unit accounting for a reduction in the *Φ*_PL_. With the reduction in the steric bulk, a higher degree of polymer backbone aggregation was likely increasing non-radiative processes.

### Upconversion photophysics

**Figure 6:**
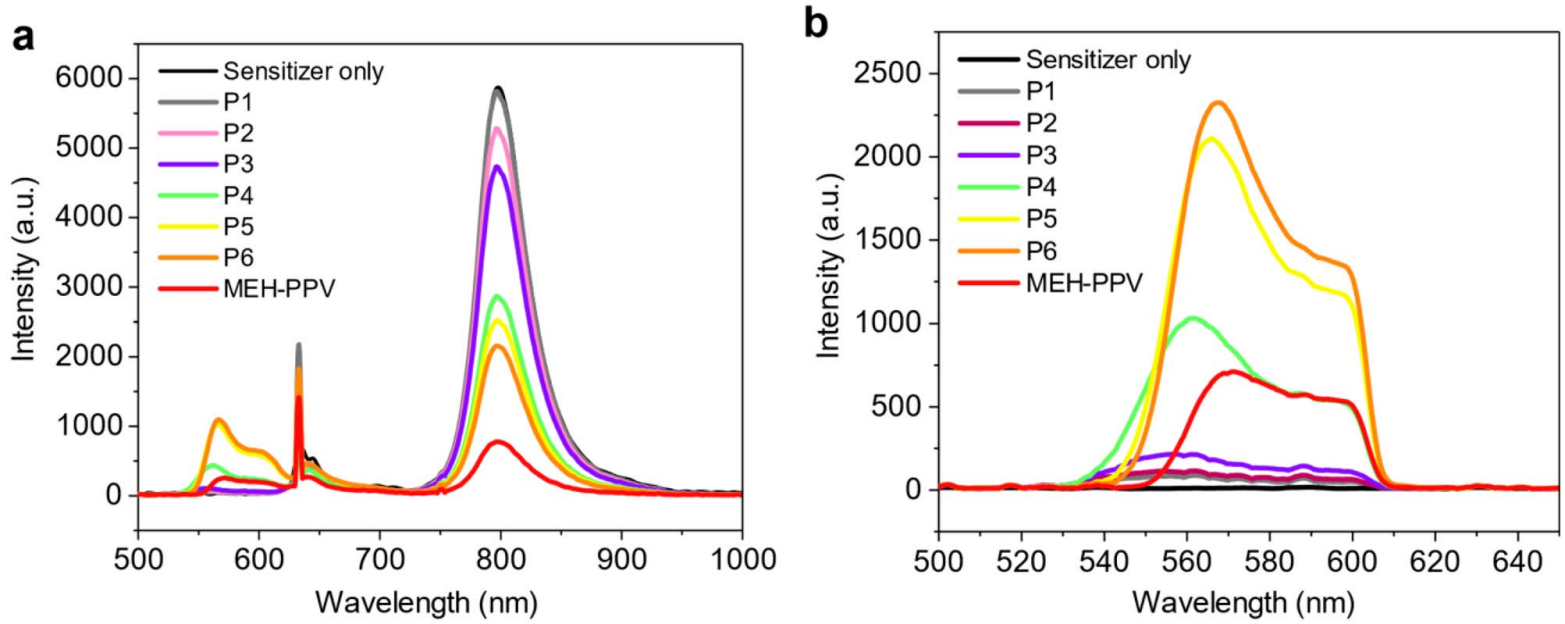
Emission spectrum of PPV copolymers **P1–P6** (0.25 mg/ml) and MEH-PPV (0.5 mg/ml) with PdTPTBP (7.5 μM) as the sensitizer in chloroform solution using 632 nm wavelength and 10,468 mW/cm^2^ power excitation without (**a**) and with (**b**) the use of a 600 nm short band pass filter. (**a**) Shows the phosphorescence emission of PdTPTBP centered at 800 nm as seen in the sensitizer-only sample, as well as the upconverted emission of the samples from 550 to 600 nm.

All the PPV copolymers showed upconversion in chloroform solution with PdTPTBP as the sensitizer (Fig. 6). A commercial sample of MEH-PPV was used as a readily available reference material (see Supplementary Information for details). The upconversion emission was optimised using **P6** by varying the polymer concentration with a fixed sensitizer concentration of 7.5 μM (Figs. S18, S19). The series of copolymers were then compared using the optimized conditions (copolymer concentration = 0.25 mg/ml), with an excitation power of 10,468 mW/cm^2^ at 632 nm. The copolymers showed a clear trend in which the quenching of the phosphorescence increased as the amount of the MEH comonomer incorporated increased, resulting in a greater upconverted emission intensity as the steric bulk decreased in moving from **P1** to **P6**. However, the resulting upconverted intensity for MEH-PPV was lower than that of many of the copolymers.

**Figure 7:**
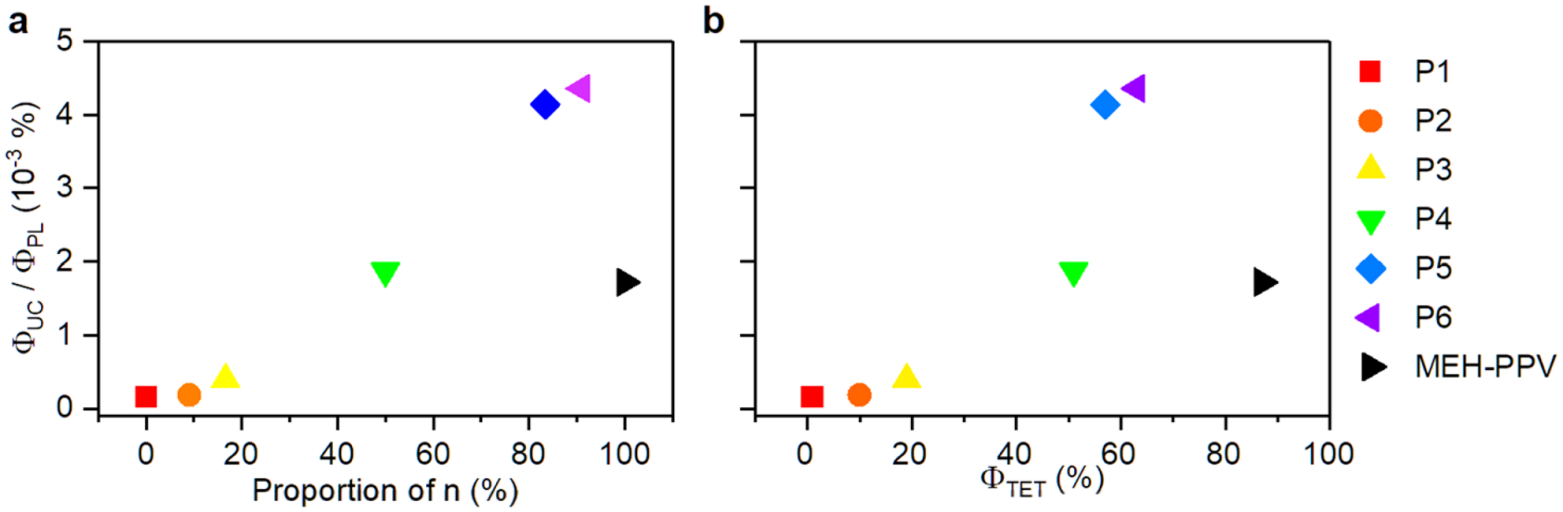
Plot of Φ_UC_/Φ_PL_ values for the PPV copolymers vs the relative proportion (in monomer units) of monomer n (**a**), plot of Φ_UC_/Φ_PL_ vs Φ_TET_ observed for each PPV copolymer (**b**).

The *Φ*_UC_ values of the copolymer increased sharply with decreasing steric bulk up until **P4** n:m = 1:5 (Fig. 7), after which a plateau is reached at **P6** n:m = 1:10, presumably due to the reduction in *Φ*_PL_. MEH-PPV has none of the bulky fluorenyloxy side chain comonomer and yet maintains a higher *Φ*_UC_ than half of the PPV copolymer**s** despite having a lower *Φ*_PL_ than the copolymers. This implied that factors other than *Φ*_PL_ in the TTA-UC process must be significantly impacting the *Φ*_UC_ (Eq. (1)). By dividing both sides of Eq. (1) by the *Φ*_PL_, the value obtained focused on all the remaining processes, specifically the TET and TTA since the ISC was the same whilst using the same sensitizer:

4 $$\frac{{\Phi }_{UC}}{{\Phi }_{PL}}={\Phi }_{ISC}{\Phi }_{TET}{\Phi }_{TTA}.$$

The role of back energy transfer (BET) from the upconverted singlet state of the conjugated polymers and the PdTPTBP sensitizer on the observed upconversion efficiency, can be determined assuming diffusional interactions. The second order diffusional rate constant for chloroform at 298 K determined from the Stokes–Einstein and Smoluchowski relationships is 5.8 × 10^9^ M^−1^ s^−1^. The concentration of PdTPTBP is 7.5 μM and, under these conditions, diffusional interactions with the polymer chains will occur on the microsecond time scale. Since the reported fluorescence lifetime of MEH-PPV, an exemplar of the conjugated polymers, is reported to be only 0.33 ns^29^, BET should not play a role in quenching the upconverted polymer singlet state.

Attempts were made to realize upconversion in solid state films following a similar procedure to Monkman et al.^24^. Using drop casting onto quartz slides from degassed solutions of the polymer and 4 wt% of the sensitizer (PdTPTBP) before being press sealed to exclude oxygen. However, no upconversion signal was detected when excited with a 632 nm laser source. This is likely due to the low efficiency so that any upconversion signal is within the low signal-to-noise regime of the detector.

Comparing the *Φ*_UC_/*Φ*_PL_ value between the PPV copolymers revealed a trend in which the value increased as the degree of steric bulk decreased (Fig. 7a). Naive extrapolation of this trend would suggest that a PPV with no side chain at all with a *Φ*_PL_ of unity would be the ideal PPV-based emitter. However, this was unrealistic as sidechains are needed to enable solution processability of the polymer as well as to suppress the aggregation-caused quenching allowing for high *Φ*_PL_ values. A similar relationship (Fig. 7b) can be seen between the *Φ*_UC_/*Φ*_PL_ value and the *Φ*_TET_ value for the polymers **P1–P6**, where *Φ*_TET_ was obtained by comparing the integrated phosphorescence of copolymer samples with the sensitizer-only sample (Table 1, see Supplementary Information for experimental details). A substantial increase in upconversion performance was observed through an increased quenching of the phosphorescence from the sensitizer in moving from **P3** to **P4** which then began to plateau above an inflection point at **P5** and **P6** such that very little improvement was made from any additional phosphorescence quenching. This phenomenon was attributed to the steric bulk of the sidechains inhibiting TET from the sensitizer to the polymer emitter, thereby reducing the quenching of sensitizers’ phosphorescence. When the copolymers consisted mostly of non-bulky monomer (m > n), the triplet energy transfer became facile, and eventually the concentration of polymer triplet excitons become favourable for TTA. Any additional triplet excitons formed may increase the likelihood of TTA events, but with diminishing returns as the polymer becomes saturated and other quenching processes that do not result in upconverted emission become more likely^30^. Interestingly, MEH-PPV shows a deviation from this observed trend attributable to a combination of low fluorescence yield and inefficient triplet triplet–triplet annihilation. The presence of the fluorenyloxy comonomer units look to be important in maintaining a significant Φ_UC_, most likely by disfavoring aggregation-induced deactivation pathways.

Using Eq. (1), the values for *Φ*_TTA_ can be obtained for the copolymer samples (Table 1). The *Φ*_TTA_ showed a generally increasing trend throughout the copolymer series with the exception of P1. There is considerable uncertainty in the *Φ*_TTA_ value for **P1** due to very low upconversion emission signal. Assuming the same *f* value for all the copolymers^9^, the contact triplet pair formation efficiency followed the same trend as *Φ*_TTA_. The assumption is reasonable since the same emitter is present in every polymer, in the form of an alkyloxy PPV backbone. The low *Φ*_TTA_ values may be attributed to triplet exciton traps decreasing the polymer triplet lifetimes or inefficient triplet–triplet annihilation processes though further spectroscopic studies will be needed to confirm this.

A further question to be considered is whether the TTA process leading to the upconverted emission is intra- or inter-molecular. An intramolecular process requires a second triplet excitation to be sensitized in a polymer chain within the lifetime of the initial triplet exciton residing on the polymer. The triplet lifetime of MEH-PPV in solution is reported to be ~ 100 microseconds^31^. The concentration of polymer chains (MEH-PPV) is 0.25 mg/ml or 1.2 × 10^–5^ M and if we assume, to a first approximation, the diffusion controlled rate of 5.8 × 10^9^ M^−1^ s^−1^ there will be polymer collisions approximately every 14 microseconds. For the very high incident laser excitation flux required to observe upconversion in these samples (~ 8 × 10^16^ photons/s including 10% reflection losses on the front cell surface at 10 W/cm^2^) and high sensitizer absorbance one might expect efficient conversion to polymer triplets at the concentrations used, assuming a diffusion-controlled collision rate. Even allowing for spin statistical factors, efficient interpolymer chain triplet–triplet annihilation and intrachain triplet–triplet annihilation to form emitting singlets are feasible. However, the very low upconversion yields observed suggests both these processes are remarkably inefficient for these polymers. Further investigations using time-resolved spectroscopies are warranted to resolve the reasons for these inefficient triplet state processes.

**Figure 8:**
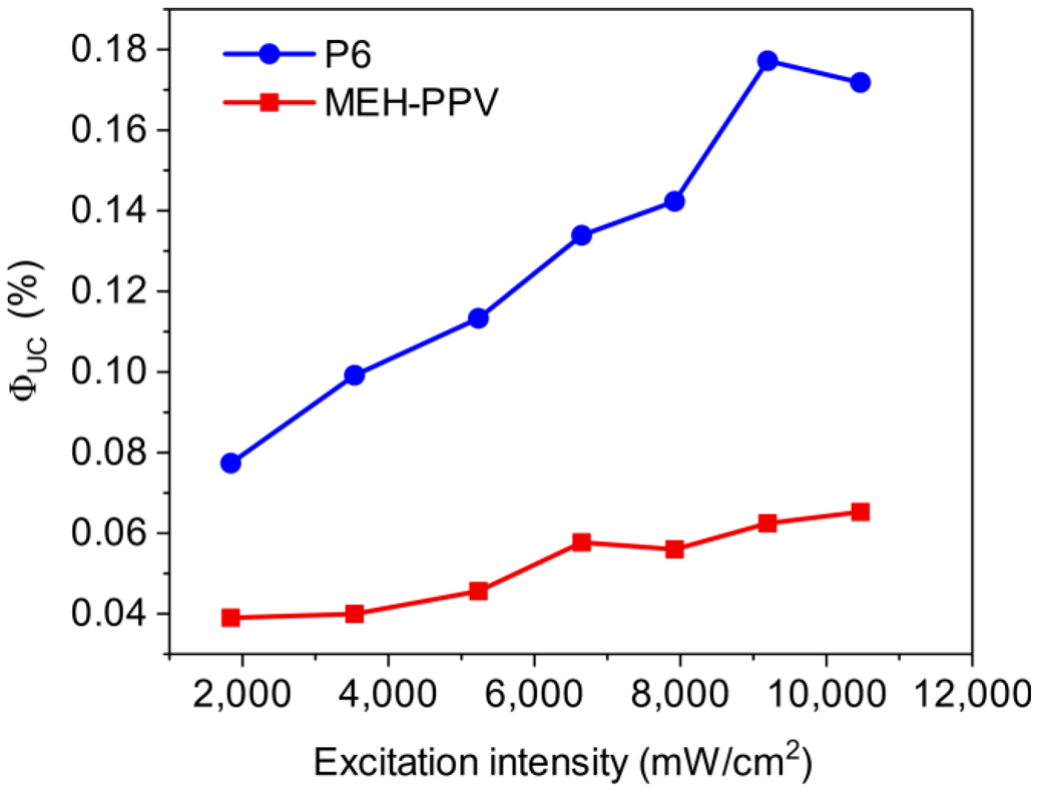
Φ_UC_ as a function of excitation intensity for copolymer **P6** and MEH-PPV.

The *Φ*_UC_ values were measured as a function of excitation intensity for both the PPV copolymer **P6** and MEH-PPV (Fig. 8). Both polymers showed an increase in *Φ*_UC_ with increasing excitation intensity up to 10,000 mW/cm^2^. This rise can be attributed to an increase in the available triplet states. The highest *Φ*_UC_ was obtained with copolymer **P6** at 9195 mW/cm^2^ with a value of 0.18%. Plotting the data on log–log scale (Fig. S22) did not reveal the upconversion excitation threshold values as both linear and quadratic relationship gave reasonable fits in the excitation intensity range. We were unable to measure *Φ*_UC_ over a greater range of excitation power due to equipment limitations.

## Conclusions

A series of PPV copolymers was synthesized to elucidate the key limiting factors of upconversion with conjugated polymers. It was found that the steric bulk on the sidechains of the polymer reduced the *Φ*_UC_ of the polymer despite increasing the *Φ*_PL_. Analysing *Φ*_UC_ and *Φ*_PL_ with respect to *Φ*_TET_ revealed sterically bulky sidechains hindered triplet energy transfer from the sensitizer to the polymer backbone. The best performing polymer was **P6** with *Φ*_UC_ of 0.18% at 635 nm excitation and 9,195 mW/cm^2^ power intensity.

## Supplementary Information

Supplementary Information.

## Data Availability

All data relevant to the discussion in this work is either shown in the manuscript or in the supporting information file. Raw data on compound characterisation and photophysical studies is available by contacting the corresponding author. The crystallographic data (CCDC 1992340) is available from The Cambridge Crystallographic Data Centre via www.ccdc.cam.ac.uk/data_request/cif.
