# Supplementary material for: Revealing the influence of steric bulk on the triplet–triplet annihilation upconversion performance of conjugated polymers
Source: Sci Rep. 2021 Oct 1;11:19585. doi: 10.1038/s41598-021-99179-y (PMC8486830; doi:10.1038/s41598-021-99179-y)
Supplement: Supplementary file 1 — Supplementary Information. [file 41598_2021_99179_MOESM1_ESM.docx]

**Supporting information**

**Revealing the influence of steric bulk on the triplet-triplet annihilation upconversion performance of conjugated polymers**

Riley O’shea,^a,b^ William J. Kendrick,^a,b^ Can Gao,^c^ Tze Cin Owyong,^a,b^ Jonathan M. White,^b^ Kenneth P. Ghiggino,^a^ Wallace W. H. Wong.^a,b^*

^a^ARC Centre of Excellence in Exciton Science, School of Chemistry, University of Melbourne, Parkville, VIC 3010, Australia

^b^Bio21 Institute, School of Chemistry, University of Melbourne, Parkville, VIC 3010, Australia

^c^Beijing National Laboratory for Molecular Sciences, Key Laboratory of Organic Solids, Institute of Chemistry, Chinese Academy of Sciences, Beijing, China

*E-mail: [wwhwong@unimelb.edu.au](mailto:wwhwong@unimelb.edu.au).

Contents

[NMR and Mass Spectra 2](#_Toc80724439)

[X-ray Crystallography 17](#_Toc80724440)

[Polymerization 19](#_Toc80724441)

[Supplementary Photophysical Data 20](#_Toc80724442)

[Measurement of absolute Φ_PL_ at high concentrations 25](#_Toc80724443)

# NMR and Mass Spectra

Figure S1 ^1^H NMR spectrum of (2-((9,9-dioctyl-9H-fluoren-2-yl)oxy)ethan-1-ol **2.**

Figure S2 ^13^C NMR spectra of (2-((9,9-dioctyl-9H-fluoren-2-yl)oxy)ethan-1-ol **2.**


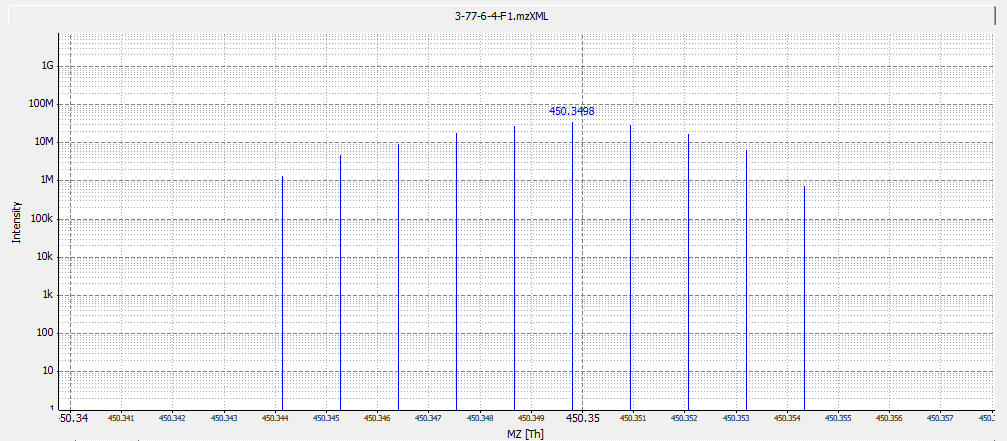


Figure S3 HRMS spectrum of (2-((9,9-dioctyl-9H-fluoren-2-yl)oxy)ethan-1-ol **2.**

Figure S4 ^1^H NMR spectra of 2-(2-bromoethoxy)-9,9-dioctyl-9H-fluorene **3.**

Figure S5 ^13^C NMR spectra of 2-(2-bromoethoxy)-9,9-dioctyl-9H-fluorene **3.**


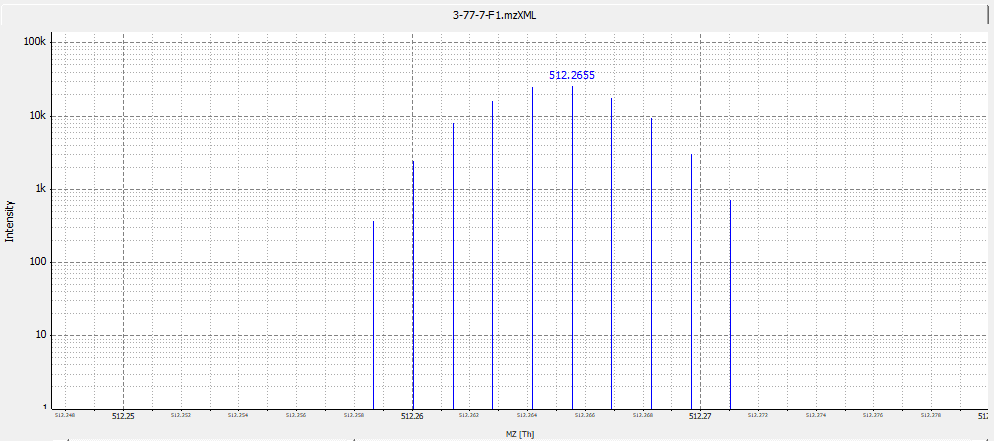


Figure S6 HRMS spectra of 2-(2-bromoethoxy)-9,9-dioctyl-9H-fluorene **3.**

Figure S7 ^1^H NMR spectra of dimethyl 2,5-bis(2-((9,9-dioctyl-9H-fluoren-2-yl)oxy)ethoxy)terephthalate **5.**

Figure S8 ^13^C NMR spectra of dimethyl 2,5-bis(2-((9,9-dioctyl-9H-fluoren-2-yl)oxy)ethoxy)terephthalate **5.**


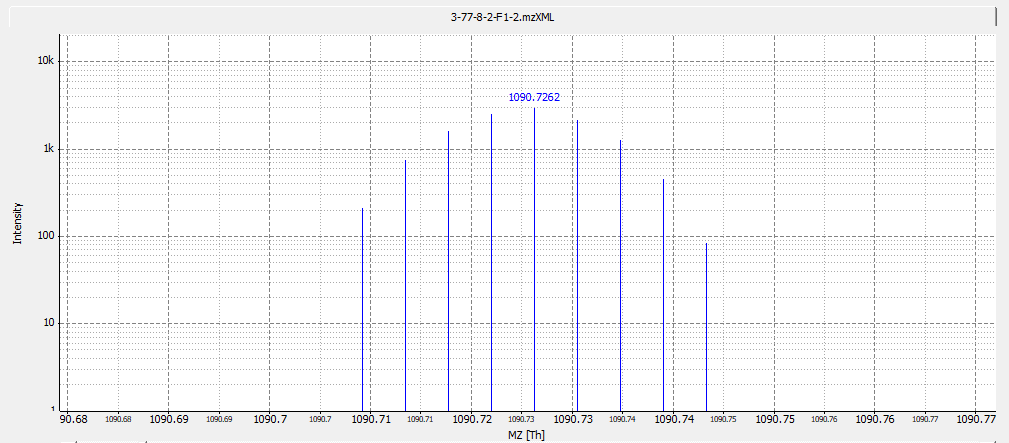


Figure S9 HRMS spectra of dimethyl 2,5-bis(2-((9,9-dioctyl-9H-fluoren-2-yl)oxy)ethoxy)terephthalate **5.**

Figure S10 ^1^H NMR spectra of (2,5-bis(2-((9,9-dioctyl-9H-fluoren-2-yl)oxy)ethoxy)-1,4-phenylene)dimethanol **6.**

Figure S11 ^13^C NMR spectra of (2,5-bis(2-((9,9-dioctyl-9H-fluoren-2-yl)oxy)ethoxy)-1,4-phenylene)dimethanol **6.**


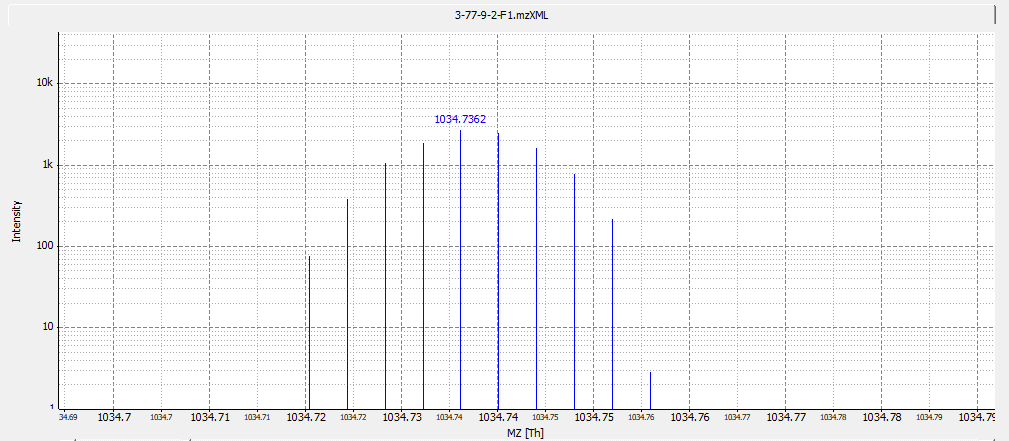


Figure S12 HRMS spectra of (2,5-bis(2-((9,9-dioctyl-9H-fluoren-2-yl)oxy)ethoxy)-1,4-phenylene)dimethanol **6.**

Figure S13 ^1^H NMR spectra of 2,2'-((((2,5-bis(chloromethyl)-1,4-phenylene)bis(oxy))bis(ethane-2,1-diyl))bis(oxy))bis(9,9-dioctyl-9H-fluorene) **7.**

Figure S14 ^13^C NMR spectra of 2,2'-((((2,5-bis(chloromethyl)-1,4-phenylene)bis(oxy))bis(ethane-2,1-diyl))bis(oxy))bis(9,9-dioctyl-9H-fluorene) **7.**


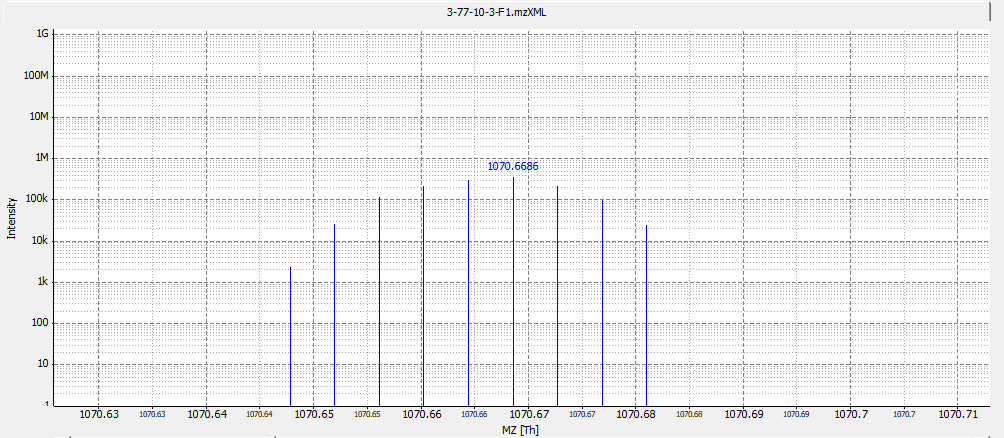


Figure S15 HRMS spectra of 2,2'-((((2,5-bis(chloromethyl)-1,4-phenylene)bis(oxy))bis(ethane-2,1-diyl))bis(oxy))bis(9,9-dioctyl-9H-fluorene) **7.**

# X-ray Crystallography

Intensity data for **7** were collected on a Rigaku Synergy Dual-source diffractometer using Cu-Kα X-radiation, the temperature during data collection was maintained at 100.0(1) K using an Oxford Cryosystems cooling device. The structure was solved by direct methods and difference Fourier Synthesis (*Acta Cryst.* **2008**, *A64*, 112.) and refined by full matrix least squares refinement on F^2^ using the WinGX software package (*J. Appl, Cryst.* **2008**, *41*, 466-470.) incorporating SHELXL-2013.(*Acta Crystallogragr. Sect. C* **2015**, *71*, 3). Thermal ellipsoid plots were generated using the Mercury software (*J. Appl. Cryst.* **2006**, *39*, 453-457).

CCDC 1992340 contains the supplementary crystallographic data for this paper. These data can be obtained free of charge from The Cambridge Crystallographic Data Centre via [www.ccdc.cam.ac.uk/data_request/cif](http://www.ccdc.cam.ac.uk/data_request/cif).

Table S1. Crystal data and structure refinement for 2,2'-((((2,5-bis(chloromethyl)-1,4-phenylene)bis(oxy))bis(ethane-2,1-diyl))bis(oxy))bis(9,9-dioctyl-9H-fluorene) **7**.

Empirical formula C_70_H_96_Cl_2_O_4_

Formula weight 1072.36

Temperature 100.00(10) K

Wavelength 1.54184 Å

Crystal system Monoclinic

Space group P 21/c

Unit cell dimensions a = 19.5864(16) Å α= 90°.

b = 17.743(2) Å β= 95.747(8)°.

c = 8.9504(6) Å γ = 90°.

Volume 3094.8(5) Å3

Z 2

Density (calculated) 1.151 Mg/m3

Absorption coefficient 1.296 mm-1

F(000) 1164

Crystal size 0.462 x 0.049 x 0.015 mm3

Theta range for data collection 3.369 to 78.315°.

Index ranges -23<=h<=24, -22<=k<=17, -10<=l<=11

Reflections collected 22615

Independent reflections 6384 [R(int) = 0.1643]

Completeness to theta = 67.684° 99.8 %

Absorption correction Semi-empirical from equivalents

Max. and min. transmission 1.00000 and 0.22841

Refinement method Full-matrix least-squares on F2

Data / restraints / parameters 6384 / 0 / 345

Goodness-of-fit on F2 1.010

Final R indices [I>2sigma(I)] R1 = 0.1095, wR2 = 0.2716

R indices (all data) R1 = 0.1862, wR2 = 0.3304

Extinction coefficient n/a

Largest diff. peak and hole 0.671 and -0.590 e.Å-3


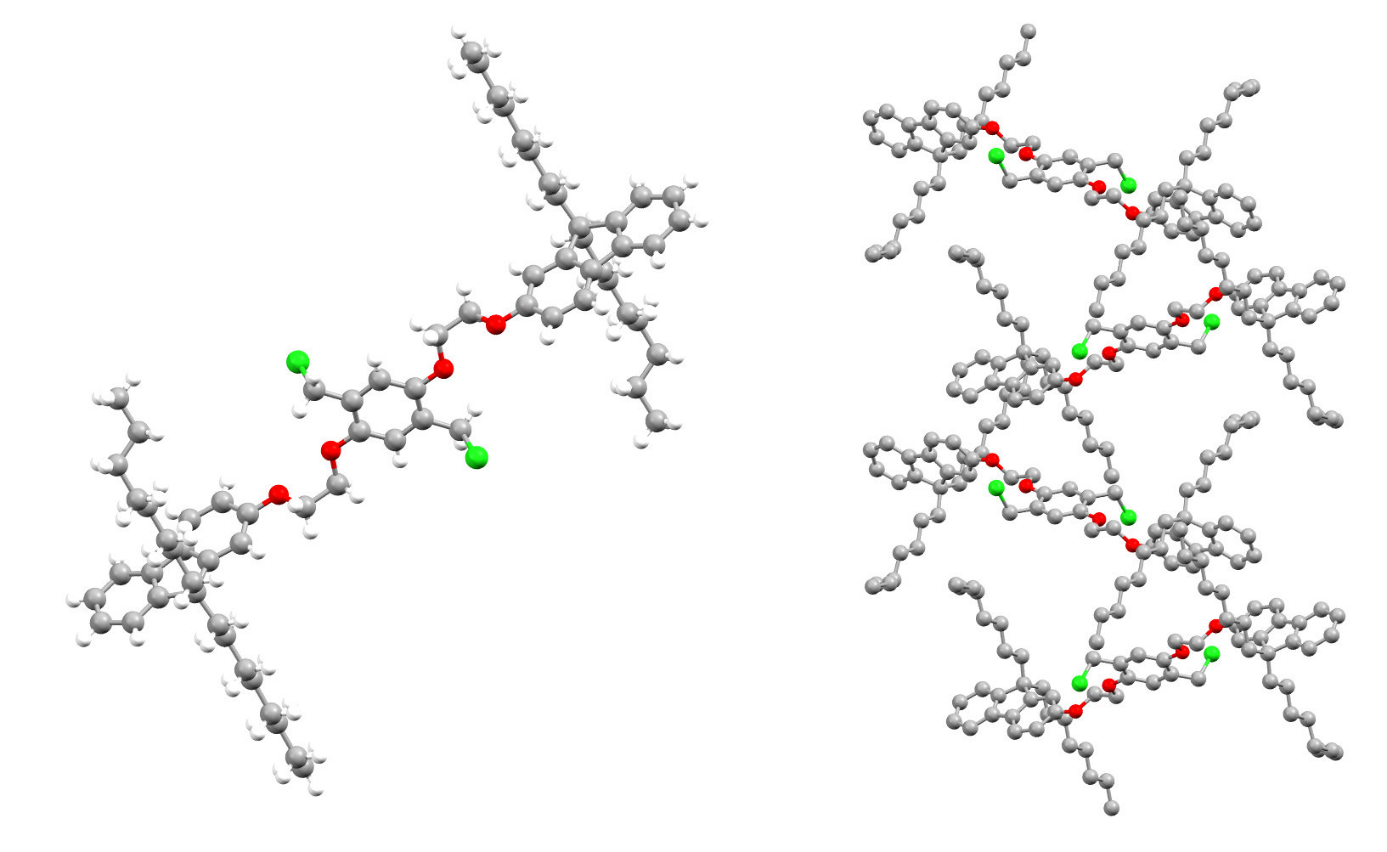


Figure S16 Single crystal structure and packing of 2,2'-((((2,5-bis(chloromethyl)-1,4-phenylene)bis(oxy))bis(ethane-2,1-diyl))bis(oxy))bis(9,9-dioctyl-9H-fluorene) **7.**

# Polymerization

Table S2 Reaction yield for PPV copolymers **P1-P6.**

| Polymer | Mass of monomer **7** (g) | Mass of monomer **8** (g) | Monomer ratio (n:m) | Polymer yield |
| --- | --- | --- | --- | --- |
| **P1** | 0.150 | 0 | 1:0 | 99% |
| **P2** | 0.141 | 0.005 | 10:1 | 57% |
| **P3** | 0.136 | 0.010 | 5:1 | 77% |
| **P4** | 0.114 | 0.045 | 1:1 | 63% |
| **P5** | 0.071 | 0.121 | 1:5 | 67% |
| **P6** | 0.049 | 0.178 | 1:10 | 68% |

Figure S17 GPC traces for the PPV copolymers **P1-6** and MEH-PPV.

# Supplementary Photophysical Data


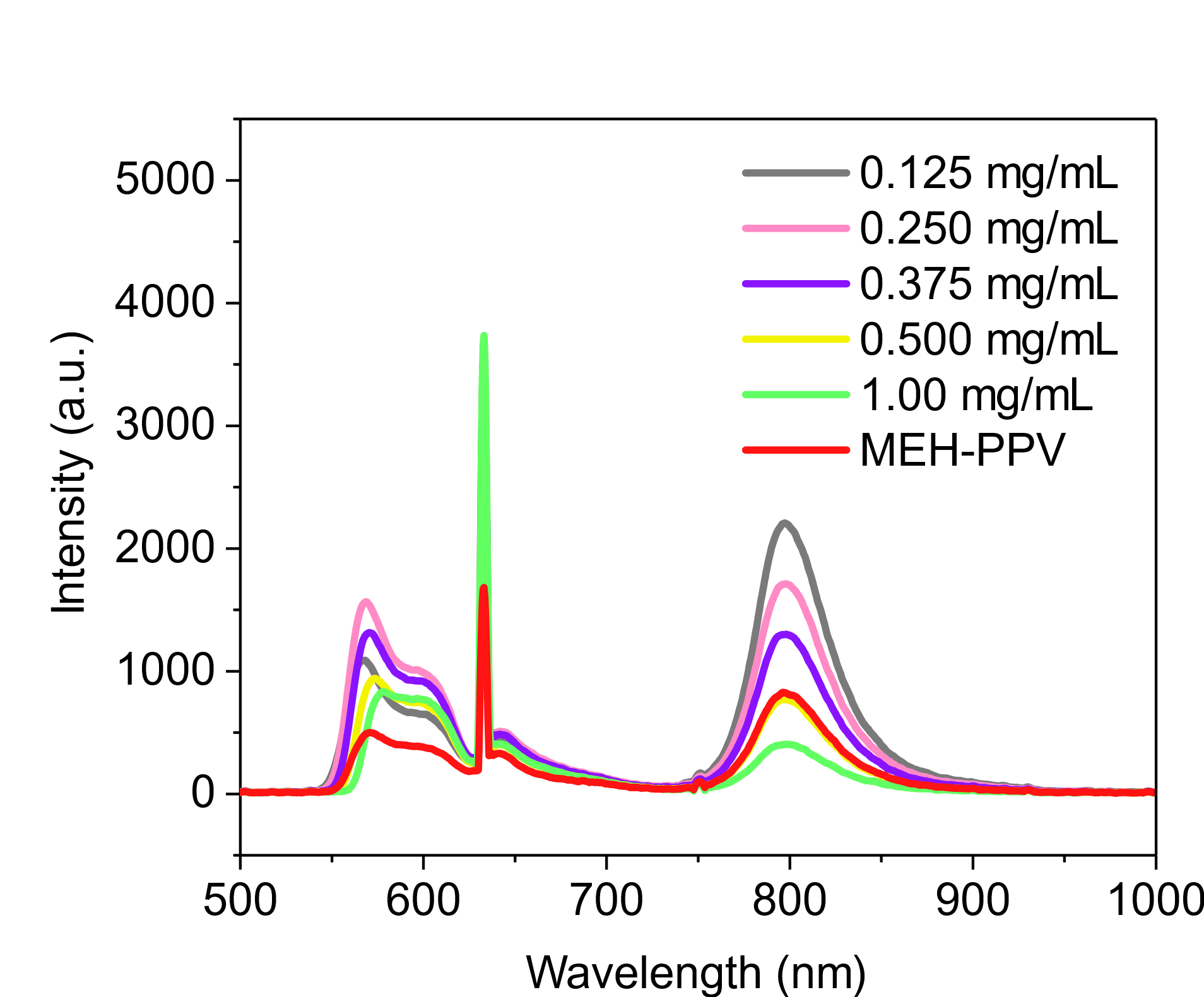


Figure S18 Photoluminescence spectra varying PPV copolymer **P6** (n:m = 1:10) concentration with fixed sensitizer (PdTPTBP) concentration = 7.5 μM, 800 ms integration time, 632 nm excitation.


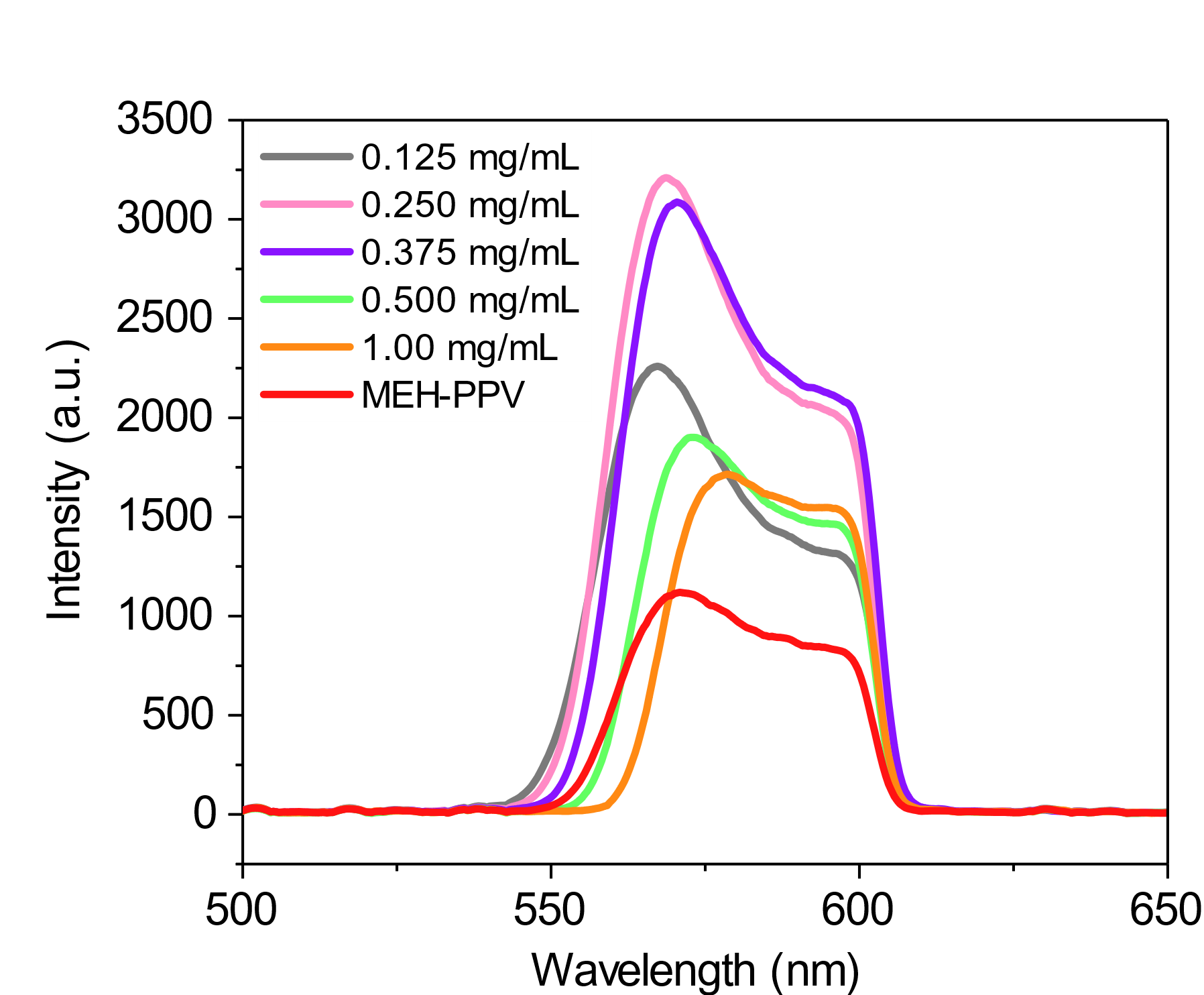


Figure S19 Photoluminescence spectra varying PPV copolymer **P6** (n:m = 1:10) concentration with fixed sensitizer (PdTPTBP) concentration = 7.5 μM, 2000 ms integration time, 632 nm excitation, with a 600 nm low band pass filter.


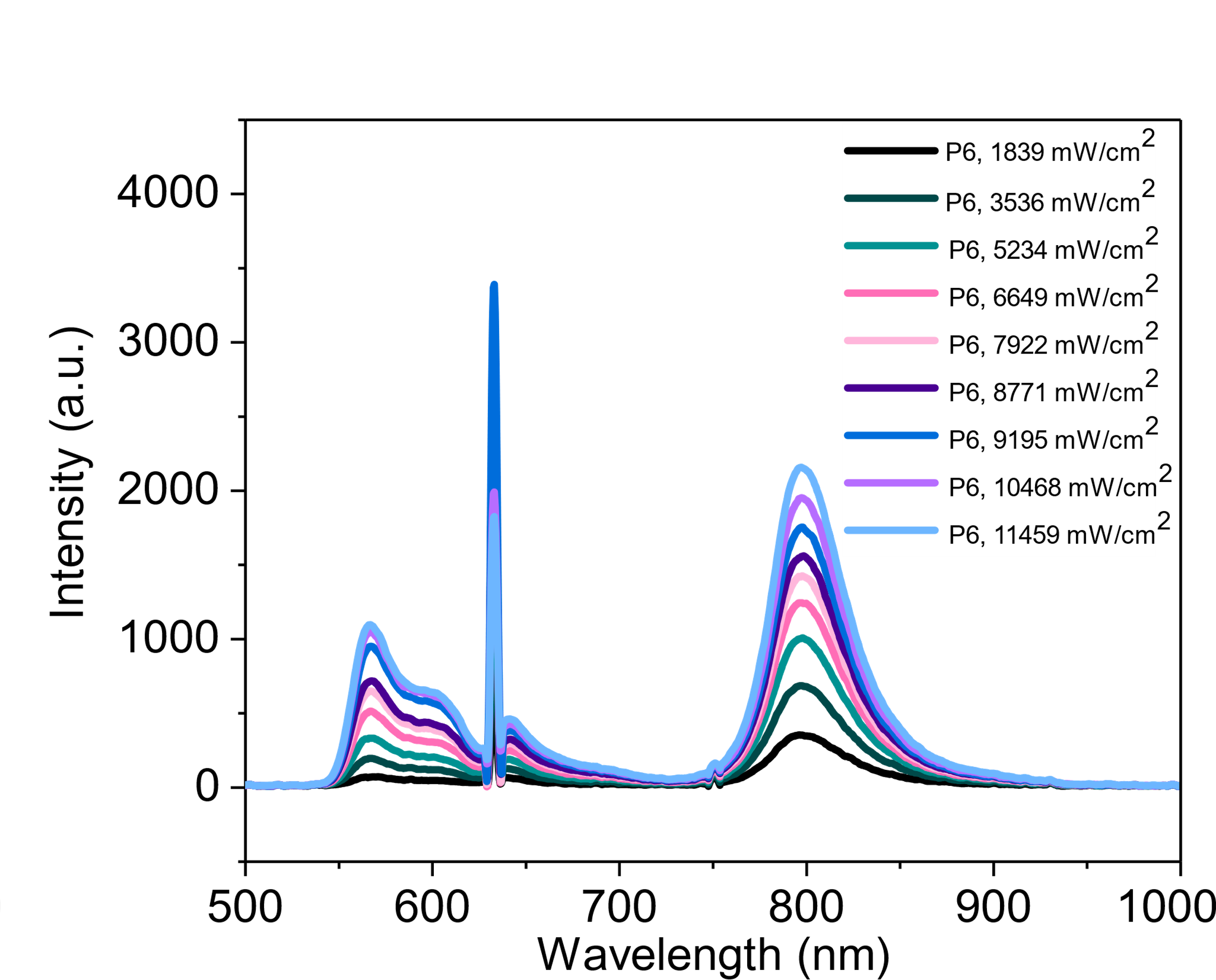


Figure S20 Photoluminescence spectra varying excitation intensity (in mW) with PPV copolymer **P6** (n:m = 1:10, 0.25 mg/mL) and PdTPTBP (7.5 μM), 800 ms integration time, 632 nm excitation, spot size = 0.28 mm^2^ (from 300 μm radius fibre optic).


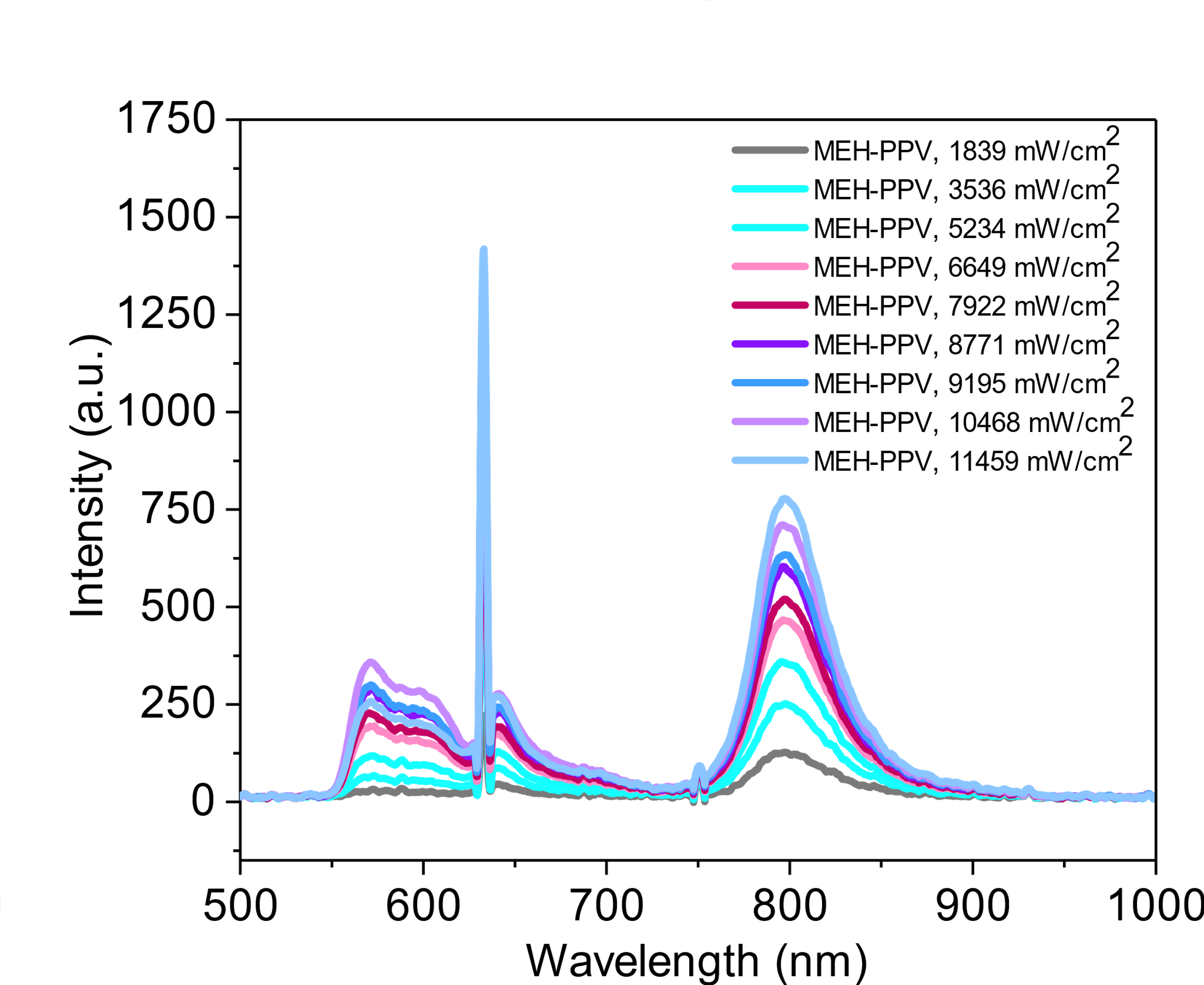


Figure S21 Photoluminescence spectra varying excitation intensity (in mW) with MEH-PPV (0.5 mg/mL) and PdTPTBP (7.5 μM), 800 ms integration time, 632 nm excitation, spot size = 0.28 mm^2^ (from 300 μm radius fibre optic).


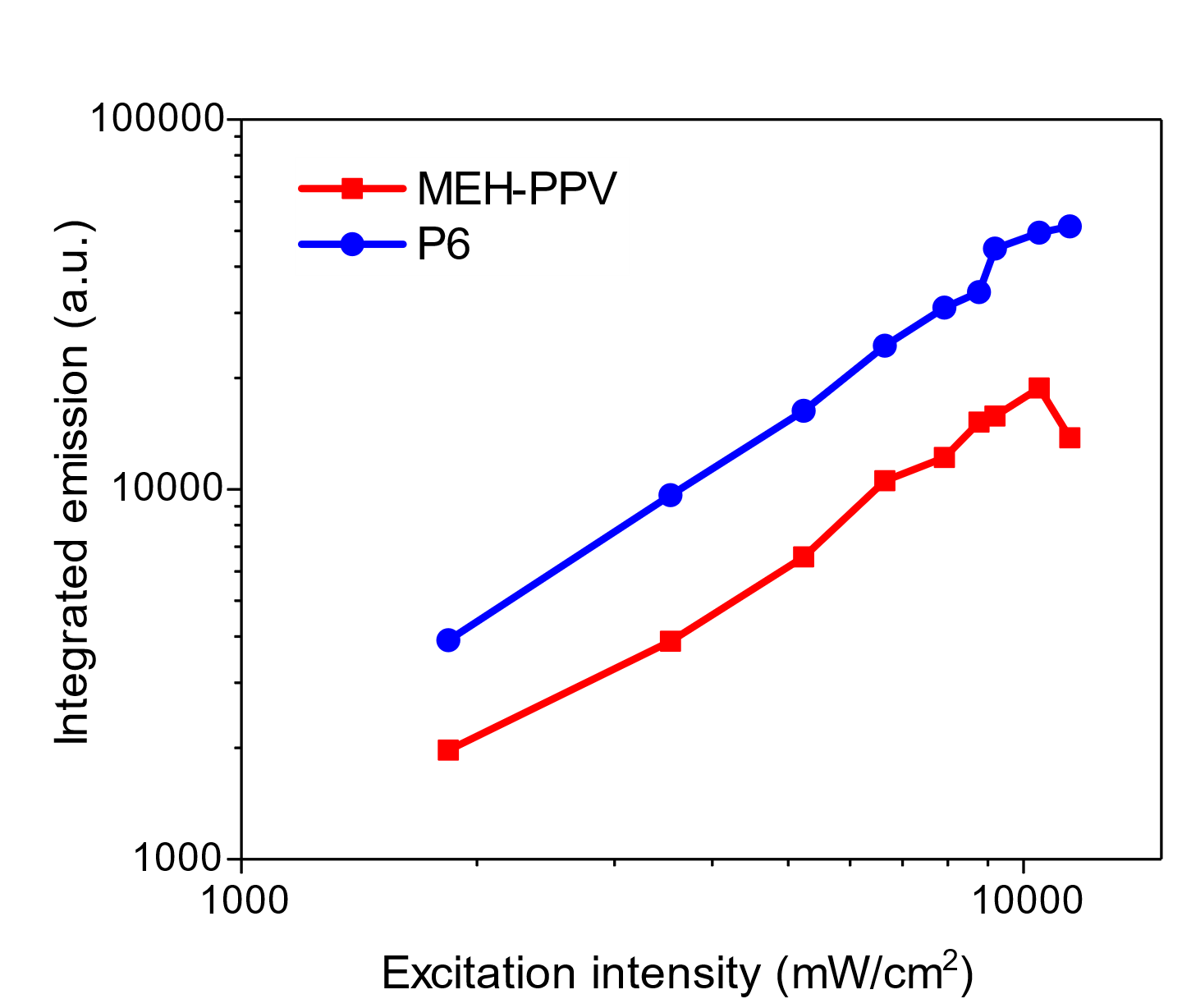


Figure S22 Log-Log plot of integrated upconversion emission vs excitation intensity for MEH-PPV (0.5 mg/mL) and copolymer **P6** (0.25 mg/mL), PdTPTBP (7.5 μM), 800 ms integration time, 632 nm excitation.


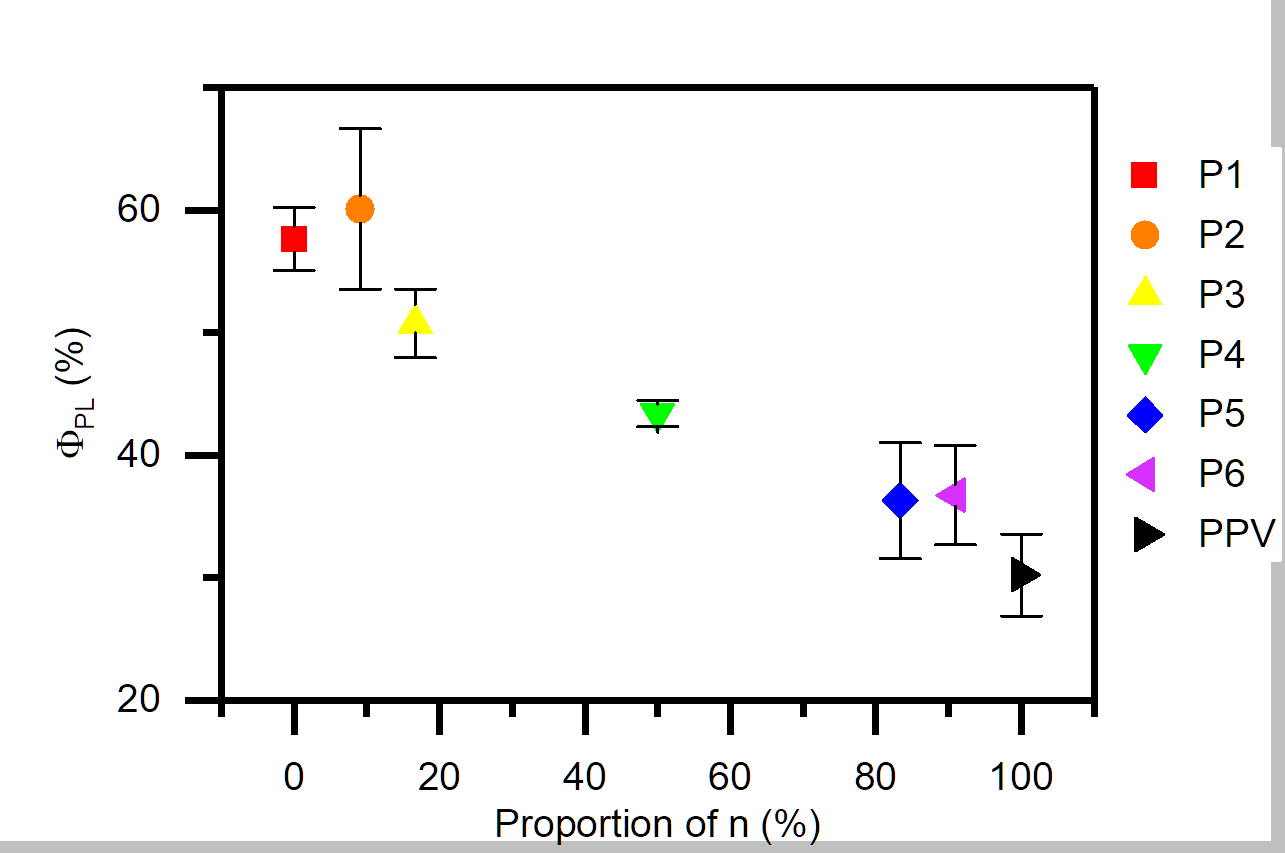


Figure S23 Plot of Φ_PL_ values for the PPV copolymers vs the relative proportion (in monomer units) of monomer n, showing a decrease in Φ_PL_ as less bulky side-chains are present in the polymers.

# Measurement of absolute Φ_PL_ at high concentrations

Absolute Φ_PL_ were measured using an integrating sphere set-up at the same concentrations used for the upconversion experiments (**P1-P6** = 0.25 mg/mL, **PPV**= 0.5 mg/mL) via the method detailed by Porrès *et al.* (*J. Fluoresc.* **2006**, *16*, 267–273.) to ensure consistency throughout the measured Φ_X_ values.

Concentrated samples are prone to reabsorption of fluorescence, which can make measurement of Φ_PL_ difficult. To minimize any reabsorption effects, the recorded integrating sphere spectra were corrected via the method presented by Würth *et al.* (*Nat. Protoc.* **2013**, *8*, 1535–1550). Reabsorption-free spectra were first recorded at the high concentrations by using a triangular optical cell in a front-facing fluorescence measurement. The spectra recorded in the integrating sphere were then matched with the reabsorption-free spectra at higher λ unaffected by reabsorption (Figure S24). Both the front-facing fluorescence measurements and the integrating sphere measurements were performed on the same day and using the same spectrometer to account for any differences between spectrometers and their associated spectral corrections.

Figure S24 Plot of the emission recorded in the integrating sphere (black), the tail-matched front-facing fluorescence spectra (red) and absorption spectra (dashed) for each of the PPV copolymers.
